# Transient coating of γ-Fe_2_O_3_ nanoparticles with glutamate for its delivery to and removal from brain nerve terminals

**DOI:** 10.3762/bjnano.11.122

**Published:** 2020-09-10

**Authors:** Konstantin Paliienko, Artem Pastukhov, Michal Babič, Daniel Horák, Olga Vasylchenko, Tatiana Borisova

**Affiliations:** 1 Palladin Institute of Biochemistry of National Academy of Sciences of Ukraine, Leontovicha Str. 9, Kyiv, 01030, Ukraine; 2 Institute of Macromolecular Chemistry AS CR, Heyrovského nám. 2, 162 06 Prague, Czech Republic; 3 National Aviation University, Liubomyra Huzara ave. 1, Kyiv, 03058, Ukraine

**Keywords:** blood plasma, brain nerve terminals, glutamate biocoating, maghemite (γ-Fe_2_O_3_) nanoparticles, protein biocorona

## Abstract

Glutamate is the main excitatory neurotransmitter in the central nervous system and excessive extracellular glutamate concentration is a characteristic feature of stroke, brain trauma, and epilepsy. Also, glutamate is a potential tumor growth factor. Using radiolabeled ʟ-[^14^C]glutamate and magnetic fields, we developed an approach for monitoring the biomolecular coating (biocoating) with glutamate of the surface of maghemite (γ-Fe_2_O_3_) nanoparticles. The nanoparticles decreased the initial rate of ʟ-[^14^C]glutamate uptake, and increased the ambient level of ʟ-[^14^C]glutamate in isolated cortex nerve terminals (synaptosomes). The nanoparticles exhibit a high capability to adsorb glutamate/ʟ-[^14^C]glutamate in water. Some components of the incubation medium of nerve terminals, that is, 4-(2-hydroxyethyl)-1-piperazineethanesulfonic acid (HEPES) and NaH_2_PO_4_, decreased the ability of γ-Fe_2_O_3_ nanoparticles to form a glutamate biocoating by about 50% and 90%, respectively. Only 15% of the amount of glutamate biocoating obtained in water was obtained in blood plasma. Albumin did not prevent the formation of a glutamate biocoating. It was shown that the glutamate biocoating is a temporal dynamic structure at the surface of γ-Fe_2_O_3_ nanoparticles. Also, components of the nerve terminal incubation medium and physiological fluids responsible for the desorption of glutamate were identified. Glutamate-coated γ-Fe_2_O_3_ nanoparticles can be used for glutamate delivery to the nervous system or for glutamate adsorption (but with lower effectiveness) in stroke, brain trauma, epilepsy, and cancer treatment following by its subsequent removal using a magnetic field. γ-Fe_2_O_3_ nanoparticles with transient glutamate biocoating can be useful for multifunctional theranostics.

## Introduction

Glutamate is a main fast excitatory neurotransmitter in the central nervous system. Normal brain function in a majority of its aspects involves glutamate signaling. The extracellular glutamate level is low between episodes of exocytotic release under normal physiological conditions, thereby preventing continual activation of glutamate receptors and protecting neurons from excitotoxic injury [1]. Each synapse presumably has a definite individual glutamate concentration in the synaptic cleft, which varies between 1 and 20 µM under normal physiological conditions depending on the method of measurements [1–2]. Glutamate uptake by the neurons and glial cells via high-affinity plasma membrane Na^+^-dependent glutamate transporters is responsible for the maintenance of the extracellular glutamate concentration. The transporters use Na^+^/K^+^ electrochemical gradients across the plasma membrane to accomplish glutamate transport.

Glutamate is a potential growth factor in tumor development. The extracellular glutamate concentrations in glioma cell lines in vitro was shown to be up to 500 µM, and glutamate stimulates glioma cell proliferation in vivo. Also, glial tumor cells ex vivo generate neurotoxic quantities of glutamate [3–6]. Excessive extracellular glutamate concentrations of 100 μM were found at the tumor margin in glioblastoma-bearing patients, which resulted in neuronal cell death and facilitated tumor growth [5,7–8]. In addition, abnormal glutamate transport and extracellular homeostasis contribute to neuronal dysfunction and are associated with the pathogenesis of major neurological disorders. Excessive ambient glutamate concentration is a characteristic feature of, among others, stroke, brain trauma, epilepsy, and seizure development.

Superparamagnetic γ-Fe_2_O_3_ nanoparticles are very promising in targeted drug delivery, cancer therapy, diagnostics, and hyperthermia treatment due to their magnetism and chemical stability [9–13]. Among a variety of other nanoparticles, superparamagnetic iron oxide nanoparticles are used for magnetic resonance imaging in cancer theranostics and magnetic hyperthermia [9–11,14]. Controlled magnetic fields can lead to induced drug release from nanoparticles to manipulate neuronal cells [9,15]. Release of receptor agonists and antagonists from thermally sensitive magnetoliposomes loaded with iron oxide magnetic nanoparticles can be remotely controlled by weak alternating magnetic fields facilitating the modulation of behavior in mice by activating ligand–receptor pairs [16]. Jia at al. used nanoparticles to modulate focal ischemia through controlling infarct size and occlusion duration by micromagnet-mediated aggregation [12].

Functionalization and vectorization of nanoparticles can be performed both by noncovalent grafting of biomolecules via ionic bonding or adsorption and by the covalent conjugation of biomolecules via strong chemical bonding [17–18]. Noncovalent nanoparticle functionalization is relatively easy to undertake. However, the results are difficult to control and to reproduce presumably because of their instability in biological media where the nanoparticles may lose their biological coating [19]. The organic/inorganic agents form a shell (1–5 nm thick) around superparamagnetic iron oxide nanoparticles interacting with their surface functional groups [14].

Sousa et al. studied the chemisorption of glutamate and aspartate by chemically modified γ-Fe_2_O_3_ nanoparticles. The glutamate and aspartate solutions were prepared in HNO_3_ at pH 3.0 and under nitrogen flux. The chemisorption was investigated in acidic medium by measuring the changes in the conductivity of the solution during addition of the ligands. Raman and FTIR spectroscopy measurements showed that glutamate and aspartate salts were adsorbed at the surface of γ-Fe_2_O_3_ nanoparticles [20]. It should be noted that the methodological protocol of above experiments on glutamate and aspartate chemisorption suggested the use of bio-aggressive and non-physiological solutions. Hence, the results cannot be translated directly to biological applications.

The aims of this study were: (i) to develop new approach of glutamate adsorption measurements in different media using radiolabeled ʟ-[^14^C]glutamate and scintillation counting; (ii) to analyze the adsorption of glutamate on γ-Fe_2_O_3_ nanoparticles forming a biocoating and, vice versa, the loss of the glutamate biocoating in a standard salt solution and physiological fluids; and (iii) to assess the effects of the nanoparticles on key characteristics of glutamatergic neurotransmission in rat brain terminals (synaptosomes). Glutamate biocoating of γ-Fe_2_O_3_ nanoparticles for glutamate delivery to and removal from the nerve terminals and other cells can be very promising in the treatment of stroke (damaged core and penumbra zones), brain trauma, epilepsy, and cancer.

## Experimental

### Synthesis of superparamagnetic maghemite (γ-Fe_2_O_3_) nanoparticles

γ-Fe_2_O_3_ nanoparticles were synthesized and characterized according to [21]. A 0.5 M solution of NH_4_OH (subaliquot amount needed for quantitative formation of Fe(OH)_3_) was added dropwise to a 0.5 M aqueous solution of FeCl_3_ under sonication (450 Digital Sonifier; Branson Ultrasonics, Danbury, CT, USA) at room temperature. Consequently, an aqueous 0.2 M solution of FeCl_2_ was added under the same conditions dropwise to Fe(OH)_3_ preformed in the first step. The mixture was then poured directly into 0.5 M NH_4_OH under nitrogen flushing and moderate stirring. The forming black suspension was left to maturate for 45 min still under stirring and nitrogen flushing. The black precipitate of magnetite (Fe_3_O_4_) was then repeatedly washed with Milli-Q water with the help of magnetic separation (approx. 10×) until spontaneous deflocculation was achieved. In the next step, the magnetite colloid was stabilized with 0.1 M sodium citrate solution under sonication. The formation of maghemite was completed by oxidation of the magnetite colloid with 5% sodium hypochlorite solution and a subsequent sonication for 5 min. The final washing of the maghemite particles was performed as described above and was also finished with 5 min of sonification. The resulting maghemite colloid of was filtered through sterile Millex syringe filters.

### Particle characterization by transmission electron microscopy

Morphology and size distribution of the particles were investigated by transmission electron microscopy (TEM; JEOL JEM 200 CX). The number-average diameter (*D*_n_) was calculated by the measurement of at least 800 particles from different microphotographs of the same sample using the IMAGEJ program. *D*_n_ = Σ*N_i_D_i_*/Σ*N_i_*, where *N_i_* is the number of particles with the diameter *D_i_*. The dispersity (*Ð*) is expressed by the ratio *D*_w_/*D*_n_, where *D*_w_ is the weight-average particle diameter, *D*_w_ = Σ*N_i_D_i_*^4^/Σ*N_i_D_i_*^3^. The hydrodynamic diameter *D*_h_ (*z*-average) and polydispersity index (PI) as measures of the distribution width were obtained by dynamic light scattering (DLS) with a ZetaSizer Nano ZS (Malvern Instruments, Malvern, UK).

### Ethics statement

Wistar rats, males, with a body weight of 250–300 g, were kept in animal facilities of the Palladin Institute of Biochemistry, National Academy of Sciences of Ukraine, as described in [22] according to the European guidelines, international laws, and policies. Rats were housed in a temperature-controlled room (22–23 °C) and provided with water and dry food pellets ad libitum. Rats were decapitated before brain removing. All experimental procedures were conducted according to standard ethical guidelines (European Community Guidelines on the Care and Use of Laboratory Animals 86/609/EEC) and the experimental protocols were approved by the Animal Care and Use Committee of the Palladin Institute of Biochemistry (Protocol from 19/09-2016). Ten animals were used in experiments analyzing ʟ-[^14^C]glutamate uptake, its extracellular level and the membrane potential of nerve terminals. Six animals were used for blood plasma preparation.

### Isolation of nerve terminals (synaptosomes) from rat cortex

The cortex zone of the rat brain was rapidly removed and homogenized in ice-cold 0.32 M sucrose, 5 mM HEPES-NaOH, pH 7.4 and 0.2 mM EDTA. The synaptosomes were prepared by differential and Ficoll-400 density gradient centrifugation of cortex homogenate in accordance to the Cotman’s method [23] with slight modifications [24]. The manipulations were performed at 4 °C. The synaptosomal suspensions were used in the experiments in a period of 2–4 h after isolation. The protein concentrations were measured in accordance to [25].

### Uptake of ʟ-[^14^C]glutamate by nerve terminals in the presence of γ-Fe_2_O_3_ nanoparticles

The uptake of ʟ-[^14^C]glutamate was recorded in the synaptosomal suspension (aliquots of 125 µL containing 0.2 mg of protein per mL) incubated in an oxygenated standard salt solution (NaCl 126 mM, KCl 10 mM, MgCl_2_ 2.0 mM, NaH_2_PO_4_ 1.0 mM, HEPES 20 mM, pH 7.4; and ᴅ-glucose 10 mM) at 37 °C for 8 min. γ-Fe_2_O_3_ nanoparticles were added to the synaptosomal suspension and further incubated for 10 min. The uptake of the neurotransmitter was started by the addition of “cold” glutamate (10 µM) supplemented with radiolabeled ʟ-[^14^C]glutamate (420 nM, 0.1 µCi/mL). The synaptosomal suspension was further incubated at 37 °C for 1 min, and then sedimented using an Eppendorf microcentrifuge at 10,000*g* for 20 s. The ʟ-[^14^C]glutamate uptake was calculated as the decrease of radioactivity in supernatant aliquots of 100 μL and the increase of radioactivity in the pellets (preliminary treated with sodium dodecyl sulfate) using liquid scintillation counting with ACS scintillation cocktail (1.0 mL) using a Delta 300 Tracor Analytic scintillation counter (USA).

### Extracellular ʟ-[^14^C]glutamate level in the nerve terminal suspension in the presence of γ-Fe_2_O_3_ nanoparticles

After preincubation in the abovementioned standard salt solution supplemented with Ca^2+^ at 37 °C for 10 min, the synaptosomal suspensions were loaded with ʟ-[^14^C]glutamate (1 nmol of protein per mg, 238 mCi per mmol) at 37 °C for 10 min. After loading, the synaptosomal suspensions were washed and the pellets were resuspended again in the standard salt solution to a final concentration of 1 mg protein per mL and immediately used in the experiments. Monitoring the extracellular ʟ-[^14^C]glutamate level was accomplished as follows: The synaptosomal suspensions (aliquots of 125 µL, containing 0.5 mg of protein per mL) were preincubated at 37 °C for 10 min. Then, γ-Fe_2_O_3_ nanoparticles were added and further incubated for 0 min and 6 min and then sedimented using an Eppendorf microcentrifuge at 10,000*g* for 20 s. Radioactivity was measured in supernatant aliquots of 100 µL using liquid scintillation counting and was expressed as a percentage of the total ʟ-[^14^C]glutamate incorporated into the nerve terminals [24].

### Membrane potential of nerve terminals in the presence of γ-Fe_2_O_3_ nanoparticles

The membrane potential was examined using rhodamine 6G (0.5 µM), a potentiometric fluorescent dye. The synaptosomal suspension at a concentration of 0.2 mg protein per mL was preincubated at 37 °C for 10 min in a stirred thermostated cuvette. In order to estimate alterations in the membrane potential, the ratio (*F*), index of the membrane potential, was calculated as *F* = *F*_t_/*F*_0_, where *F*_0_ and *F_t_* were the fluorescence intensities of rhodamine 6G in the absence and presence of nerve terminals, respectively. *F*_0_ was calculated through extrapolation of the exponential decay function to *t* = 0. The fluorescence was measured at 528 nm (excitation) and 551 nm (emission), with slit bands of 5 nm each, using a Hitachi MPF-4 spectrofluorimeter.

### Blood plasma preparations

Rat blood was drawn by cardiac puncture with plastic syringes. Heparin was used as anticoagulant (50 U per milliliter of blood). The samples of rat blood were collected into plastic tubes and rapidly centrifuged at 130*g* for 20 min, platelet-rich plasma was decanted and centrifuged at 800*g* for 10 min. The supernatant was used in the experiments.

### Assessment of adsorption capability of γ-Fe_2_O_3_ nanoparticles

Glutamate adsorption by γ-Fe_2_O_3_ nanoparticles was measured in the standard salt solution and in its separate components, that is, KCl (10 mM), NaH_2_PO_4_ (1 mM), HEPES (20 mM), and also in albumin (0.125 mg/mL) and blood plasma. An amount of 1.5 µL of ʟ-[^14^C]glutamate (500 nM, 238 mCi/mmol) and non-radiolabeled glutamate at definite concentrations were mixed with abovementioned components along with different amounts of γ-Fe_2_O_3_ nanoparticles also added to the probe. The incubation time was 5 min in the salt solutions and 30 min in blood plasma or albumin. After that a magnetic field was applied or the nanoparticles were sedimented in a microcentrifuge (10 min at 13,000*g*). Adsorption was measured in the aliquots of the supernatants (100 µL) by liquid scintillation counting with ACS scintillation cocktail (1.5 mL), expressed as percentage of the total amount of radiolabelled glutamate adsorbed. The ʟ-[^14^C]glutamate count at definite concentrations was considered as 100% in further calculations.

### Analysis of the size of γ-Fe_2_O_3_ nanoparticles in different media by laser correlation spectroscopy

The size of γ-Fe_2_O_3_ nanoparticles was measured by dynamic light scattering using a laser correlation spectrometer ZetaSizer-3 (Malvern Instrument, UK) equipped with a He–Ne laser LGN-111 (*P* = 25 mW, λ = 633 nm). A suspension of γ-Fe_2_O_3_ nanoparticles (1 mL) in the standard salt solution was placed to a cylindrical quartz cuvette of 10 mm in diameter, which was inserted into the laser correlation spectrometer. The measurement range of the instrument is from 1 nm to 50 µm. Registration and statistical processing of laser light scattered by a water (*n* = 1.33) suspension of the nanoparticles, measured repeatedly for 120 s at 22 °С at a scattering angle of 90°, were carried out. The results of the experiments were processed using the software PCS-Size mode v1.61. The laser correlation spectrometer was equipped with a multi-computing correlator type 7032 CE.

### Simulation of spatial structure of γ-Fe_2_O_3_ nanoparticles coated with blood plasma protein biocorona

Simulations of biocorona formation at the surface of γ-Fe_2_O_3_ nanoparticles were performed using software for graphic-mathematical modeling, that is, a demonstration version of Diamond 4.5.3 (Crystal Impact GbR^©^) for the simulation of nanoparticle polycrystals, a demonstration version of LeadIT 2.3.2 (BioSolveIT GmbH^©^) to find the most probable binding sites between protein and nanoparticles, a free version of ArgusLab 4.0.1 (Mark Thompson and Planaria Software LLC^©^) for modeling a biomodified nanoparticle, and a trial version of the Materials Sciences Suite 2015 (Schrödinger Software^©^) software package to generate appropriate images of the whole protein–nanoparticle cluster.

### Statistical analysis

The results were expressed as mean ± S.E.M. of *n* independent experiments. One-way ANOVA followed by post hoc Tukey’s test was used for data processing. The accepted level of significance was set as *P* < 0.05.

## Results

### Methodological approach for monitoring glutamate biocoating of γ-Fe_2_O_3_ nanoparticles in different media

**Figure 1:**
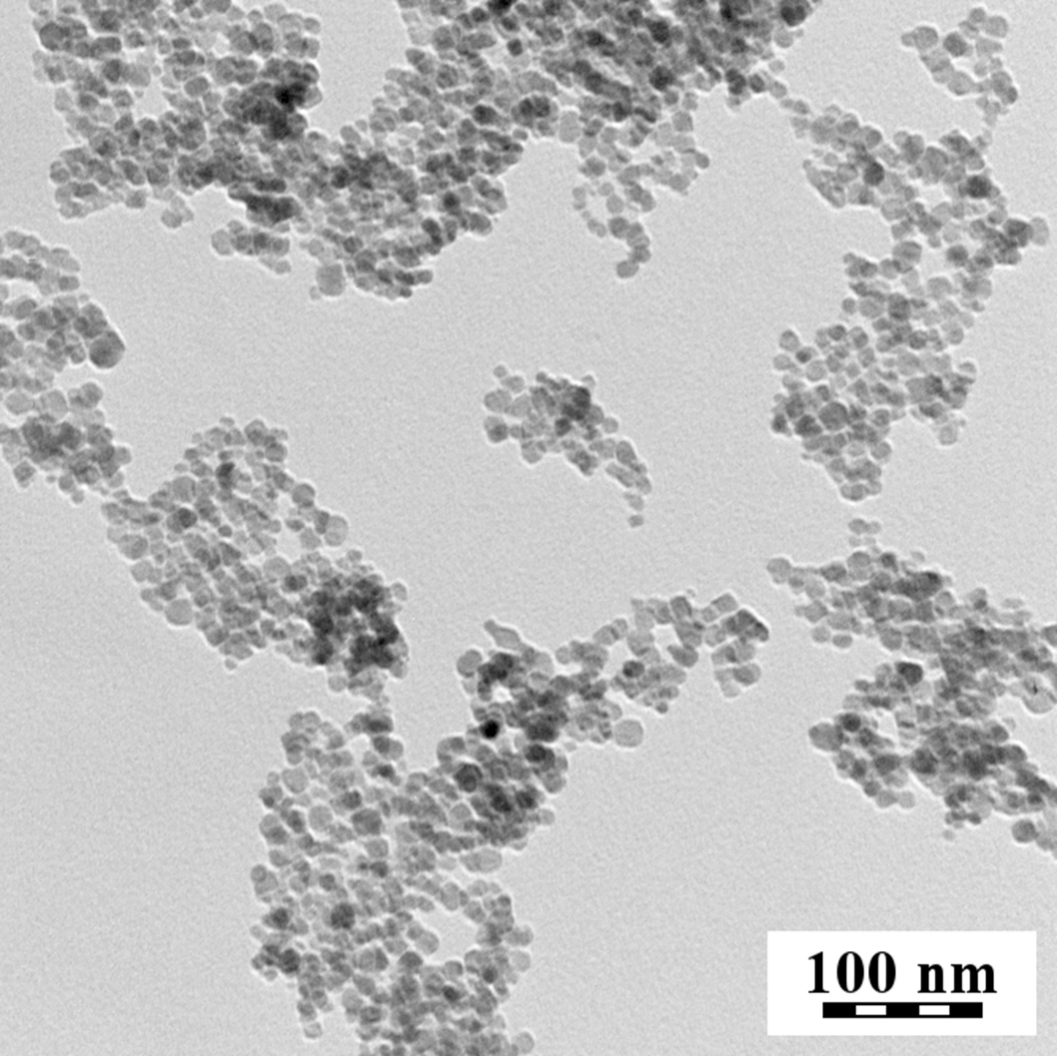
TEM micrograph of γ-Fe_2_O_3_ nanoparticles.

Nanoparticles of γ-Fe_2_O_3_ were prepared by the coprecipitation method and oxidation with sodium hypochlorite. According to TEM (dry samples, Figure 1), they have a size *D*_n_ = 9.7 nm and a dispersity *Ð* = 1.21. According to DLS, the hydrodynamic size of newly synthesized nanoparticles was *D*_h_ = 85.1 ± 0.3 nm, with a polydispersity index PI = 0.108 ± 0.006, and a zeta potential ζ = −55.2 ± 0.5 mV at pH 7.8.

Using radiolabeled ʟ-[^14^C]glutamate and liquid scintillation counting, a methodological approach was developed for a direct monitoring of the glutamate biocoating at the surface of γ-Fe_2_O_3_ nanoparticles. Also, its dynamics and stability in a standard salt solution, physiological fluids, and in the presence of certain compounds were measured. This approach allows for the evaluation of glutamate delivery to and removal from nerve terminals and blood plasma for modulation of extracellular glutamate homeostasis. For this purpose, γ-Fe_2_O_3_ nanoparticles were incubated with different amounts of ʟ-[^14^C]glutamate supplemented with cold non-radiolabeled glutamate (see Experimental section) for 5 min in water or salt solutions and for 30 min in plasma or albumin. To avoid any contribution of unbound glutamate/ʟ-[^14^C]glutamate, the γ-Fe_2_O_3_ nanoparticles were isolated from the incubation medium using a magnetic field (250 mT, gradient 5.5 Т/m). Alternatively, the nanoparticles were sedimented in a microcentrifuge (10 min at 13,000*g*). After removal of the nanoparticles from the incubation media, they were washed repeatedly with 10 volume equivalents of water. The formation of glutamate biocoating was measured as a decrease in radioactivity in the aliquots of the incubation medium (100 mL) by liquid scintillation counting with ACS scintillation cocktail (1 mL). The value of ʟ-[^14^C]glutamate radioactivity at definite concentrations in the corresponding incubation medium with no nanoparticles added was used in calculations as a control (100%) and consisted of 30,000 cpm/mL at 1.86 µM glutamate/ʟ-[^14^C]glutamate. The results were expressed as percentage of the total amount of ʟ-[^14^C]glutamate in the control.

### Efficacy of glutamate biocoating of γ-Fe_2_O_3_ nanoparticles in water

**Figure 2:**
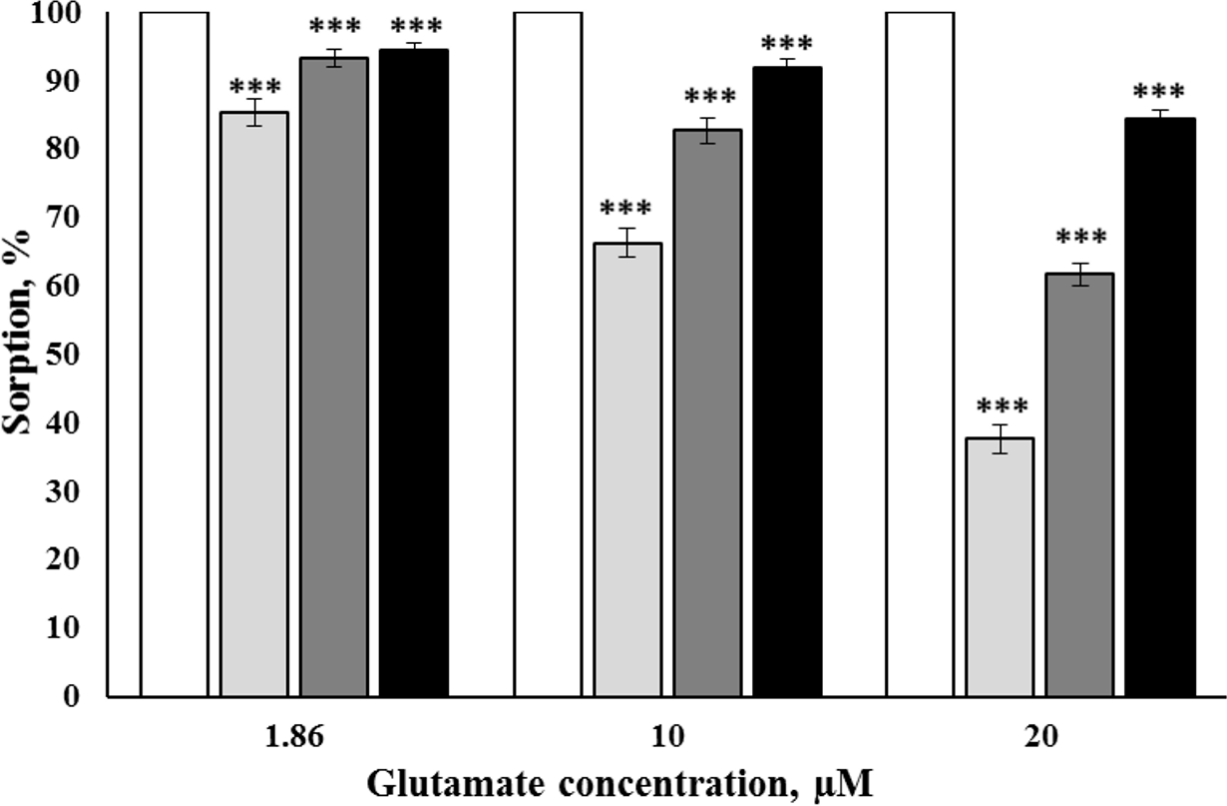
Glutamate/ʟ-[^14^C]glutamate biocoating formation at the surface of γ-Fe_2_O_3_ nanoparticles in water at a concentration of 0.75 mg/mL (the second columns in the tetrads), 1.5 mg/mL (the third columns in the tetrads), and 3 mg/mL (the fourth columns in the tetrads). The glutamate concentration was 1.86 µM (the first tetrad), 10 µM (the second tetrad), and 20 µM (the third tetrad). The ʟ-[^14^C]glutamate count at the corresponding concentrations in each tetrad was considered as 100%. Data are compared by one-way ANOVA and post hoc Tukey’s test. ***: *Р* < 0.001 compared to the corresponding control (the first columns in the tetrad).

The first sets of the experiments were aimed to assess the glutamate biocoating of γ-Fe_2_O_3_ nanoparticles in water. We have analyzed whether the concentration ratio glutamate/nanoparticles influenced the adsorption of the neurotransmitter, since the literature data have indicated that the nanoparticle concentration is a crucial parameter for the absorption efficacy [26–27]. The ʟ-[^14^C]glutamate count at the corresponding concentrations in each tetrad was considered as 100% in further calculations (Figure 2). It was shown that nanoparticles at a concentration of 0.75 mg/mL were able to adsorb 85.3% ± 2.0% of 1.86 µM glutamate/ʟ-[^14^C]glutamate, at a concentration of 1.5 mg/mL they adsorbed 93.3% ± 1.2% of 1.86 µM glutamate/ʟ-[^14^C]glutamate, and at a concentration of 3 mg/mL they adsorbed 94.4% ± 1.1% of 1.86 µM glutamate/ʟ-[^14^C]glutamate (*F*_(3,16)_ = 21.6, *Р* < 0.001, *n* = 5) (Figure 2). Nanoparticles at a concentration of 0.75 mg/mL were able to adsorb 66.3% ± 2.0% of 10 µM glutamate/ʟ-[^14^C]glutamate, at a concentration of 1.5 mg/mL they adsorbed 82.7% ± 1.9% of 10 µM glutamate/ʟ-[^14^C]glutamate, and at a concentration of 3 mg/mL they adsorbed 91.9% ± 1.2% of 10 µM glutamate/ʟ-[^14^C]glutamate (*F*_(3,16)_ = 92.2, *Р* < 0.001, *n* = 5). Nanoparticles at a concentration of 0.75 mg/mL were able to adsorb 37.7% ± 2.0% of 20 µM glutamate/ʟ-[^14^C]glutamate, at a concentration of 1.5 mg/mL they adsorbed 61.7% ± 1.7% of 20 µM glutamate/ʟ-[^14^C]glutamate, and at a concentration of 3 mg/mL they adsorbed 84.5% ± 1.3% of 20 µM glutamate/ʟ-[^14^C]glutamate (*F*_(3,16)_ = 332.6, *Р* < 0.001, *n* = 5).

### Effects of γ-Fe_2_O_3_ nanoparticles on ʟ-[^14^C]glutamate uptake and the ambient level of ʟ-[^14^C]glutamate in the brain nerve terminal preparations

**Figure 3:**
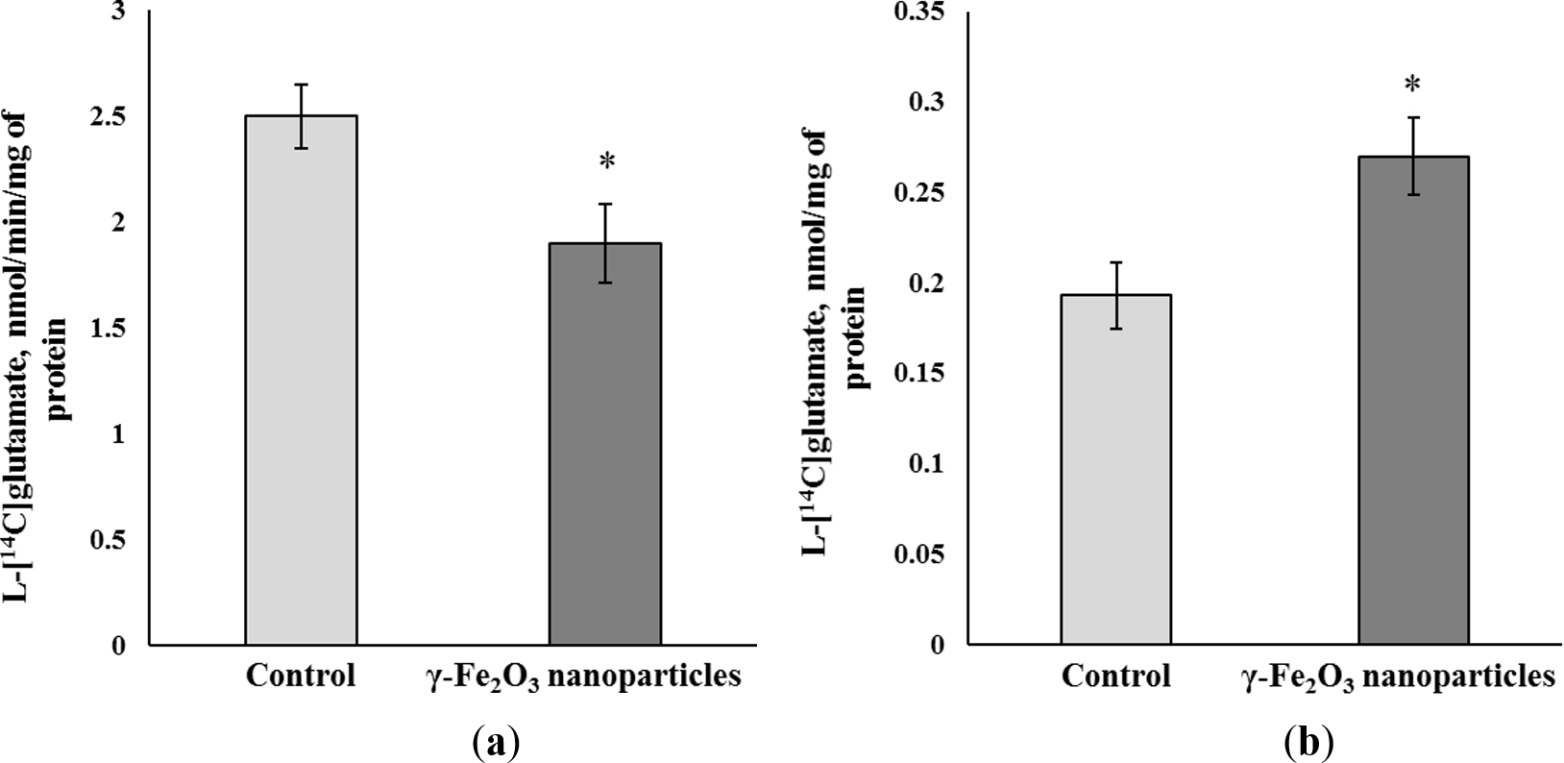
(a) The initial rate of ʟ-[^14^C]glutamate uptake and (b) the ambient level of ʟ-[^14^C]glutamate in the nerve terminal preparations in the presence of γ-Fe_2_O_3_ nanoparticles (1 mg/mL). The data are mean ± S.E.M. of four independent experiments, each performed in triplicate. The data are compared by one-way ANOVA and post hoc Tukey’s test. *: *Р* < 0.05 compared to the control.

The capability of nanoparticles to form a glutamate biocoating shown in the previous subsection can change the effectiveness of ʟ-[^14^C]glutamate uptake by isolated nerve terminals (synaptosomes). The latter is an excellent system to analyze presynaptic transport processes [1]. γ-Fe_2_O_3_ nanoparticles at a concentration of 1 mg/mL decreased the initial rate of synaptosomal ʟ-[^14^C]glutamate uptake by approximately 25% (Figure 3a). The initial rate was equal to 2.50 ± 0.15 nmol/min/mg of protein in the control and decreased to 1.90 ± 0.18 nmol/min/mg of protein in the presence of γ-Fe_2_O_3_ nanoparticles (*F*_(1,6)_ = 6.5 , *Р* < 0.05, *n* = 4).

If a decrease in the uptake was a result of ʟ-[^14^C]glutamate adsorption by γ-Fe_2_O_3_ nanoparticles, it is logical that the nanoparticles can decrease also the ambient ʟ-[^14^C]glutamate concentration in the synaptosomal preparations. However, γ-Fe_2_O_3_ nanoparticles increased the synaptosomal ambient level of ʟ-[^14^C]glutamate (Figure 3b), which was 0.193 ± 0.018 nmol/mg of protein in the control and 0.270 ± 0.021 nmol/mg of protein and in the presence of γ-Fe_2_O_3_ nanoparticles at a concentration of 1 mg/mL (*F*_(1,6)_ = 7.5, *Р* < 0.05, *n* = 4). When the concentration of γ-Fe_2_O_3_ nanoparticles was decreased to 50 and 1 µg/mL, no significant changes were revealed in the ambient level of ʟ-[^14^C]glutamate in nerve terminal preparations. The levels were 0.195 ± 0.017 and 0.210 ± 0.019 nmol/mg of protein, respectively. Hence, only at a high concentration of 1 mg/mL did the nanoparticles influence the initial rate of ʟ-[^14^C]glutamate uptake and the ambient level of ʟ-[^14^C]glutamate in the nerve terminal preparations.

### Effects of γ-Fe_2_O_3_ nanoparticles on the membrane potential of nerve terminals

**Figure 4:**
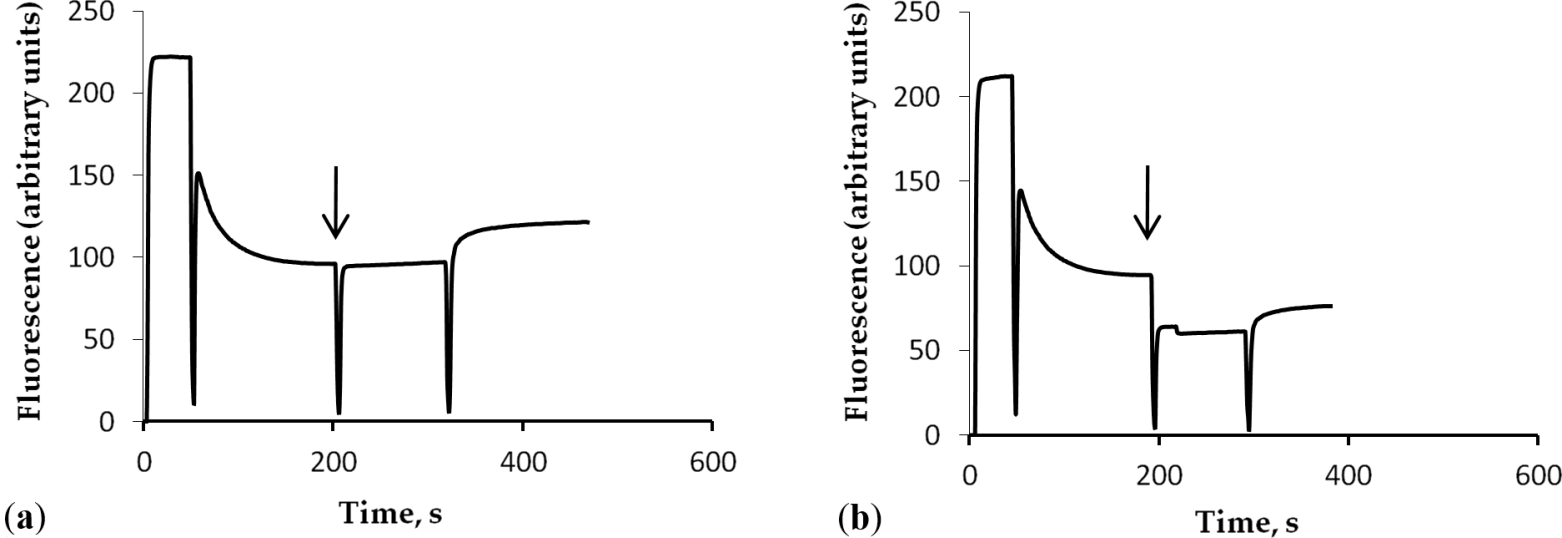
The synaptosomal membrane potential after the addition of γ-Fe_2_O_3_ nanoparticles. The synaptosomal suspension was equilibrated with rhodamine 6G (0.5 µM); when the steady-level of the dye fluorescence was reached, γ-Fe_2_O_3_ nanoparticles at a concentration of (a) 1 µg/mL and (b) 50 µg/mL were added (arrows). Each trace represents four experiments performed with different preparations.

The plasma membrane potential (*E*_m_) characterizes the capability of nanoparticles to disturb the integrity of nerve terminals [2]. The measurements were carried out using the potential-sensitive fluorescent dye rhodamine 6G, which binds to negatively charged membranes. The addition of synaptosomes to the dye-containing medium was accompanied by a partial decrease in fluorescence signal due to rhodamine 6G binding to the synaptosomal plasma membrane. First, the membrane potential index at the steady-state level was reached after a period of 3 min. As shown in Figure 4, the application of γ-Fe_2_O_3_ nanoparticles at a concentration of 1 µg/mL did not change the fluorescence signal of the dye. An increase in the nanoparticle concentration up to 50 µg/mL changed the fluorescence intensity of rhodamine 6G, which was associated with a decrease in transparency of the analyzed solution. In the fluorescence experiments, a γ-Fe_2_O_3_/synaptosomal protein ratio similar to that used in the experiments regarding uptake and extracellular level of ʟ-[^14^C]glutamate cannot be used because of poor probe transparency. Hence, in this set of the experiments lower concentrations of γ-Fe_2_O_3_ nanoparticles were used. It was demonstrated that γ-Fe_2_O_3_ nanoparticles at low concentrations did not influence the fluorescence intensity of rhodamine 6G in the synaptosomal preparations, which in turn reflected an absence of membrane depolarization in the nerve terminals.

### Glutamate biocoating of γ-Fe_2_O_3_ nanoparticles in the presence of different components of synaptosomal incubation medium

The question rose from the above experiments, whether or not glutamate biocoating at the surface of the nanoparticles was a reason of changes in synaptosomal uptake and the ambient level of ʟ-[^14^C]glutamate. It is expected from ʟ-[^14^C]glutamate biocoating formation experiments (Figure 2) that the ʟ-[^14^C]glutamate transport parameters (Figure 3) can be distorted by nanoparticle-mediated ʟ-[^14^C]glutamate adsorption. However, uptake and ambient level results were not adequate. Based on the literature data that carboxylic acids adsorbed on the oxide surface form a chelate-type bonds involving Fe(III) surface species and a carboxylate group [24], it was suggested that certain components of the synaptosomal incubation medium, for instance, chelating agents from the buffer solution, can prevent glutamate biocoating of the nanoparticles.

**Figure 5:**
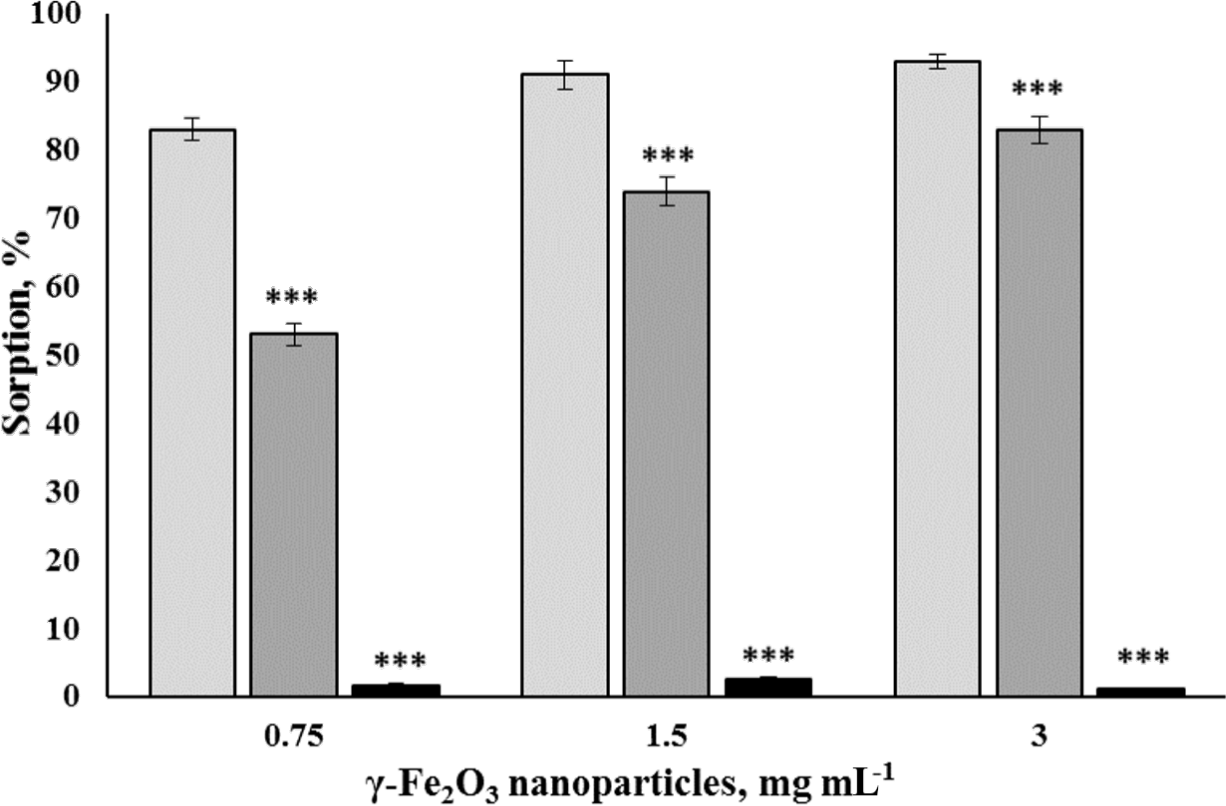
Glutamate/ʟ-[^14^C]glutamate biocoating formation at the surface of γ-Fe_2_O_3_ nanoparticles at a concentration of 0.75 mg/mL (the first triplet of columns), 1.5 mg/mL (the second triplet of columns), and 3 mg/mL (the third triplet of columns) in the presence of 10 mM KCl (the first columns in the triplets), 20 mM HEPES (the second columns in the triplets), and 1 mM NaH_2_PO_4_ (the third columns in the triplets). The glutamate/ʟ-[^14^C]glutamate concentration was 1.86 µM. The data are compared by one-way ANOVA and post hoc Tukey’s test. ***: *Р* < 0.001 as compared to KCl data.

The components of the standard salt solution (10 mM KCl, 20 mM HEPES and 1 mM NaH_2_PO_4_) were tested whether they prevent the formation of the glutamate/ʟ-[^14^C]glutamate biocoating at the nanoparticle surface. As shown in Figure 5, 10 mM KCl had no effect on the ability of γ-Fe_2_O_3_ nanoparticles, at concentrations of 0.75, 1.5, and 3 mg/mL, to adsorb 1.86 µM glutamate/ʟ-[^14^C]glutamate compared to an aqueous solution. Also, an almost complete inhibition of glutamate/ʟ-[^14^C]glutamate biocoating at the nanoparticle surface was shown with 1 mM NaH_2_PO_4_. In particular, at a nanoparticle concentration of 0.75 mg/mL, the adsorption of 1.86 µM glutamate/ʟ-[^14^C]glutamate was 83.0% ± 1.7% in 10 mM KCl, 53.0% ± 1.6% in 20 mM HEPES, and 1.6% ± 0.3% in 1 mM NaH_2_PO_4_ (*F*_(2,9)_ = 943.4, *Р* < 0.001, *n* = 4), respectively. At a nanoparticle concentration of 1.5 mg/mL, the adsorption of 1.86 µM glutamate/ʟ-[^14^C]glutamate was 91.0% ± 2.0% in 10 mM KCl, 74.0% ± 2.1% in 20 mM HEPES, and 2.5% ± 0.5% in 1 mM NaH_2_PO_4_ (*F*_(2,9)_ = 761.4, *Р* < 0.001, *n* = 4), respectively. At a nanoparticle concentration of 3 mg/mL, the adsorption of 1.86 µM glutamate/ʟ-[^14^C]glutamate was 93.0% ± 1.0% in 10 mM KCl, 83.0% ± 1.9% in 20 mM HEPES, and 1.1% ± 0.2% in 1 mM NaH_2_PO_4_ (*F*_(2,9)_ = 1544.3, *Р* < 0.001, *n* = 4).

A dramatic decrease and almost complete elimination of the glutamate biocoating occurred in the presence of phosphate ions. Also, HEPES affected the adsorption of glutamate/ʟ-[^14^C]glutamate on the nanoparticle surface, but to a lesser extent.

It was confirmed by these adsorption experiments that changes in synaptosomal ʟ-[^14^C]glutamate uptake and the ambient level were not associated with the adsorption of glutamate/ʟ-[^14^C]glutamate by the nanoparticles during the experiments. No coating with glutamate/ʟ-[^14^C]glutamate occurred at the surface of the nanoparticles during uptake and ambient level measurements in the synaptosomal incubation medium.

### Distribution of γ-Fe_2_O_3_ nanoparticles by intensity in different media measured using laser correlation spectroscopy

**Figure 6:**
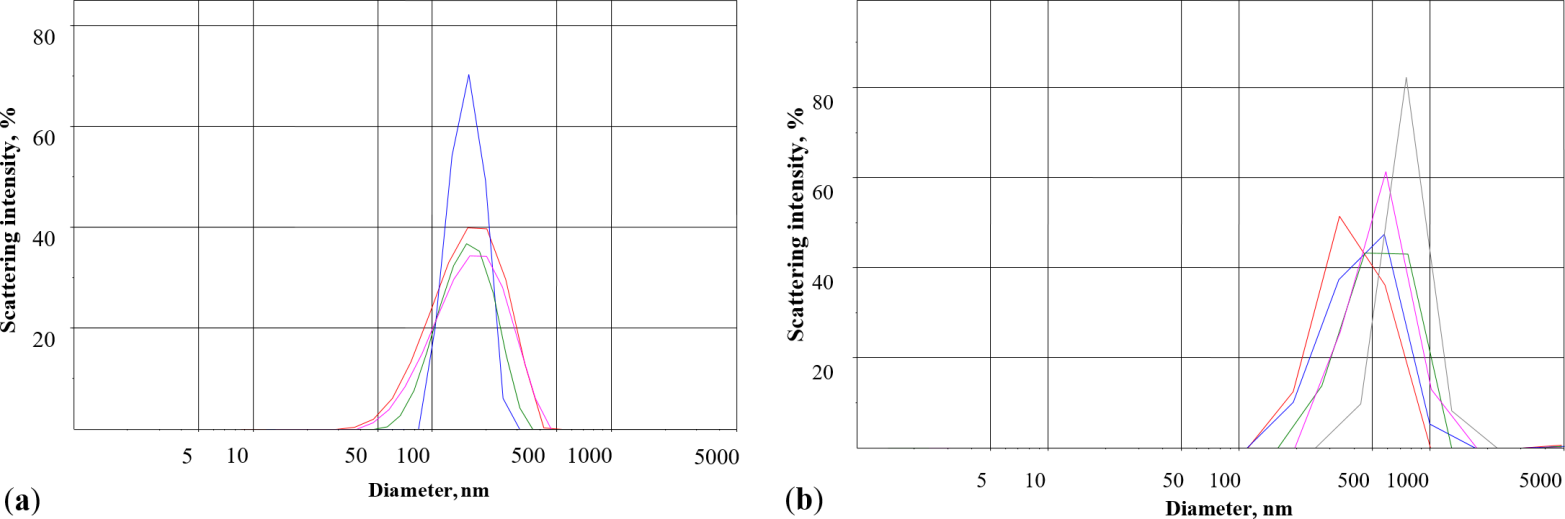
Laser correlation spectroscopy diagrams. Distribution of γ-Fe_2_O_3_ nanoparticles (0.5 mg/mL) by intensity in (a) water and (b) the standard salt solution containing the chelating agents HEPES and NaH_2_PO_4_. Each measurement was carried out over a period of 1 min. Red line: first measurement, blue line: second measurement, green line: third measurement, violet line: fourth measurement, grey line: fifth measurement.

Nanoparticle distribution by intensity was measured using dynamic light scattering. Peak analysis (intensity) revealed that the overall mean was 171 nm in water (Figure 6a), which is a higher value than that according to the TEM micrographs (10 nm) [21]. This results from the difference of fundamentals of both methods and the possible spontaneous aggregation of ferrimagnetic iron oxide particles. The nanoparticle distribution was also analyzed in the standard salt solution (synaptosomal incubation medium). After addition of the nanoparticles to the medium containing chelating agents, such as HEPES and NaH_2_PO_4_, their average size significantly increased and the mean size value was 564 nm (Figure 6b). HEPES and Na_2_HPO_4_ changed the distribution of the nanoparticles and increased their average size. This is in agreement with measurements of glutamate/ʟ-[^14^C]glutamate-coated nanoparticles, where the HEPES and Na_2_HPO_4_ chelating agents prevented glutamate adsorption (Figure 5).

### Effects of blood plasma and albumin on the formation of a glutamate biocoating on γ-Fe_2_O_3_ nanoparticles

**Figure 7:**
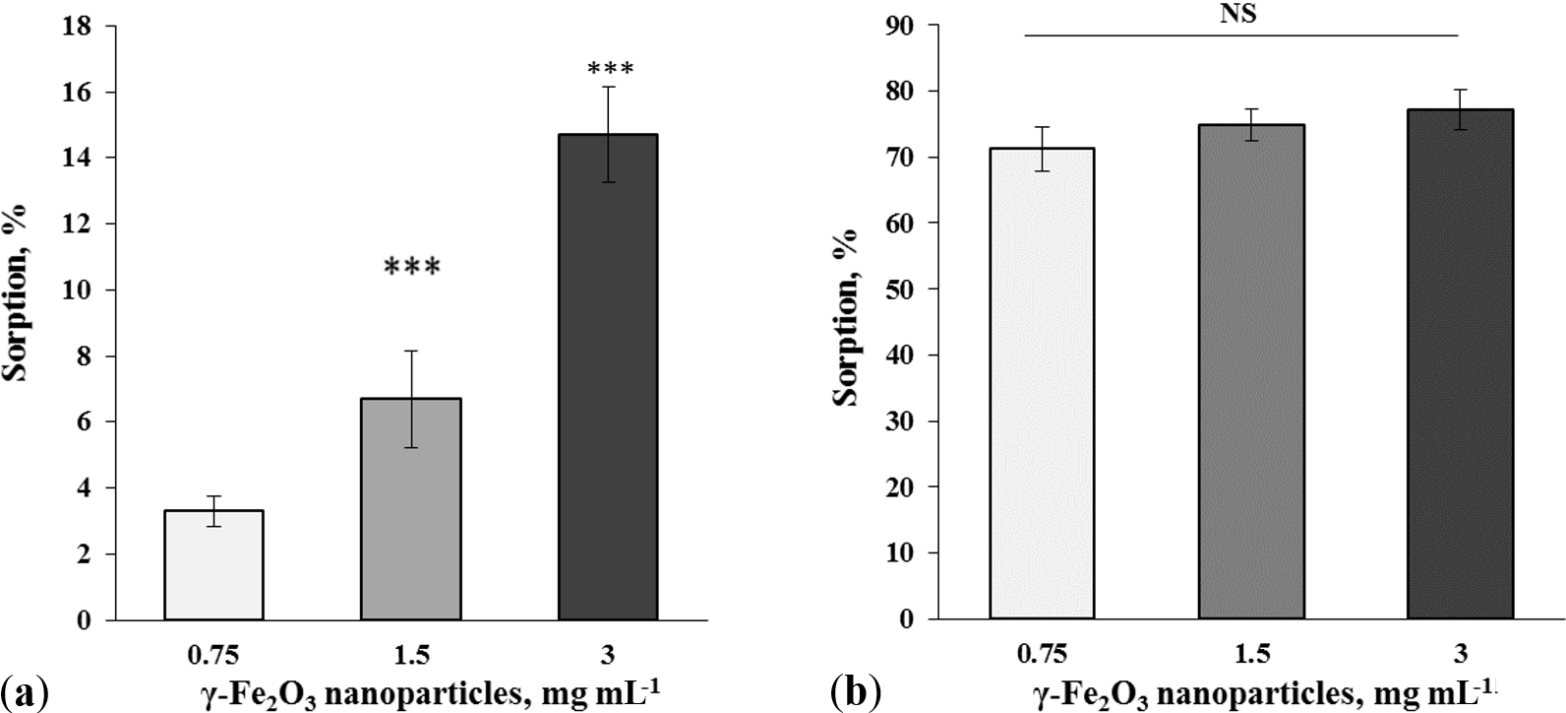
Glutamate/ʟ-[^14^C]glutamate biocoating at the surface of γ-Fe_2_O_3_ nanoparticles at concentrations of 0.75 mg/mL (the first column), 1.5 mg/mL (the second column), and 3 mg/mL (the third column) in the presence of (a) blood plasma and (b) albumin. The glutamate/ʟ-[^14^C]glutamate concentration was 1.86 µM. The data are compared by one-way ANOVA and post hoc Tukey’s test. ***: *Р* < 0.001 compared to 0.75 mg/mL data; NS: no statistically significant difference.

In this set of the experiments, the glutamate/ʟ-[^14^C]glutamate biocoating at the surface of γ-Fe_2_O_3_ was monitored in blood plasma. It was revealed that blood plasma significantly decreased the adsorption ability of the nanoparticles. The nanoparticles at concentrations of 0.75, 1.5 and 3 mg/mL adsorbed 3.3% ± 0.5%, 6.7% ± 1.5% and 14.7% ± 1.4% of 1.86 µM glutamate/ʟ-[^14^C]glutamate, respectively, in the presence of blood plasma (*F*_(2,9)_ = 23.1, *Р* < 0.001, *n* = 4) (Figure 7a). Therefore, a preliminary treatment of γ-Fe_2_O_3_ nanoparticles with blood plasma reduced the glutamate biocoating availability by more than 75%. Albumin is the main protein component of blood plasma with a concentration of 45 mg/mL. To analyze whether proteins or other chelating components of blood plasma affected the glutamate/ʟ-[^14^C]glutamate adsorption, albumin was applied at a concentration of 0.125 mg/mL (Figure 7b). It was revealed that albumin added before glutamate/ʟ-[^14^C]glutamate application had no effect on glutamate adsorption by the nanoparticles (*F*_(2,9)_ = 1.0, *Р* > 0.05, *n* = 4). Therefore, not albumin, but other components of blood plasma caused a decrease in the glutamate biocoating capability of γ-Fe_2_O_3_ nanoparticles.

### Distribution of γ-Fe_2_O_3_ nanoparticles by intensity in the presence of blood plasma measured using laser correlation spectroscopy

**Figure 8:**
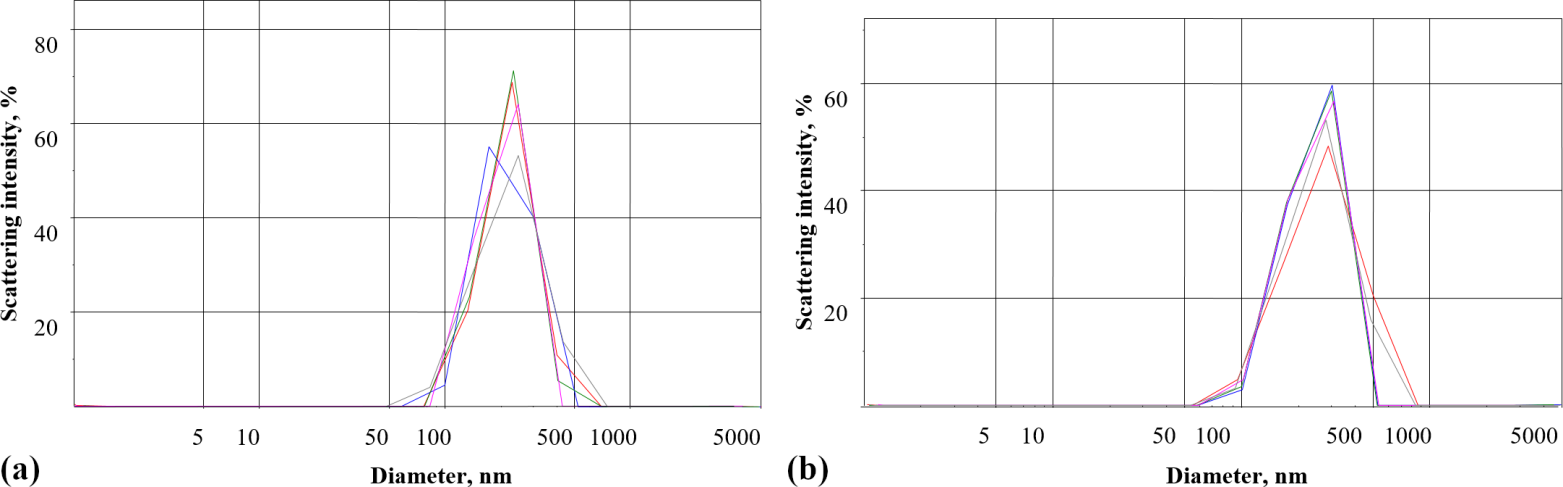
Laser correlation spectroscopy diagrams. Distribution of γ-Fe_2_O_3_ nanoparticles (0.5 mg/mL) by intensity in (a) blood plasma and (b) after preliminary incubation with blood plasma followed by transfer into the standard salt solution. Each measurement was carried for a period of 1 min. Red line: first measurement, blue line: second measurement, green line: third measurement, violet line: fourth measurement, grey line: fifth measurement.

The nanoparticle distribution by intensity was measured using dynamic light scattering in the presence of blood plasma (Figure 8a). Peak analysis (intensity) revealed that the mean nanoparticle size was 220 nm, which was insignificantly larger than the nanoparticle size in water (Figure 6a), but significantly smaller than that in the standard salt solution (Figure 6b). It was analyzed whether or not blood plasma can prevent a nanoparticle size increase caused by chelating components of the standard salt solution. In order to do so, the nanoparticles after treatment with blood plasma were transferred into the standard salt solution. The mean nanoparticle size was 245 nm (Figure 8b), confirming that blood plasma prevented a significant increase in particle size due to presence of chelating agents in the standard salt solution.

### Simulation of the spatial structure of a maghemite nanoparticle coated with blood plasma protein biocorona

Interaction of the nanoparticles and plasma proteins was also analyzed with computer modeling and simulations of spatial structures using the programs LeadIT 2.3.2, ArgusLab 4.0.1 and Material Science Suite 2015, and the online Protein Data Bank (PDB) service (see Supporting Information File 1) [28–32].

## Discussion

The conditions in biological systems can alter the properties of nanoparticles. Salts of biological fluids influence surface charges, which leads to destabilization and aggregation of nanoparticles [18]. It is well-known that upon entrance into biological systems nanoparticles interact with macromolecules of physiological fluids and tissues forming a biocorona at the surface. Macromolecules, such as proteins, nucleic acids, and lipids, become associated with different types of nanoparticles. The biocorona can consist of hundreds of proteins, which influence the physical and chemical properties of the nanoparticles, such as, size, morphology, aggregation state, hydrophobicity, and permeability. Hence, it is difficult to predict the behavior of nanoparticles in biological systems [13,18,33]. Ho et al. studied how biomolecules influenced the stability of gold nanoparticles and showed that serum proteins at high concentrations stabilized the nanoparticles, whereas lower concentrations enhanced nanoparticle aggregation. Also, immunoglobulins and fibrinogen caused a greater extent of particle aggregation than other studied proteins [27]. Zhang et al. analyzed the effects of different concentrations of nanoparticles on the zeta potential in serum protein media, and demonstrated that 5% fetal bovine serum can cover almost the whole surface of nanoparticles at a concentration below 500 µg/mL. The zeta potential of the nanoparticles was determined by the proteins located at the nanoparticle surface. In contrast, the sign of the nanoparticle charge could be reversed when the nanoparticle concentration was 1000 µg/mL. These data indicated that the serum proteins did not cover the whole surface of the nanoparticles [26]. Abakumov et al. revealed that nanoparticles coated with bovine serum albumin can be used for glioma visualization and drug delivery of anticancer therapeutics [34]. Analysis of the biocoating formation at the surface of nanoparticles is crucial for understanding the mechanisms of nanoparticle interaction with biological cells. It was demonstrated that besides the formation of a macromolecular biocorona, nanoparticles can interact with small bioactive molecules of physiological fluids and tissues forming biocoatings at the surface. Glutamate can form chemical bonds with the metal ions present at the γ-Fe_2_O_3_ nanoparticle surface [20].

In this study, new approach for monitoring the formation and the stability of a glutamate biocoating at the surface of γ-Fe_2_O_3_ nanoparticles was developed using radiolabeled ʟ-[^14^C]glutamate and physiologically compatible media. The possibility of modulating the glutamate transport in nerve terminals and blood plasma was analyzed. It was revealed that 90% of glutamate/ʟ-[^14^C]glutamate was bound to the nanoparticles in water (Figure 2). The chelating agents HEPES and NaH_2_PO_4_, the components of Krebs Ringer salt solution and synaptosomal incubation medium, decreased the ability of γ-Fe_2_O_3_ nanoparticles to form a glutamate biocoating by 50% and 90%, respectively (Figure 5). Data on the absence of glutamate/ʟ-[^14^C]glutamate adsorption at the nanoparticle surface in synaptosomal incubation medium were confirmed by the experiments with nerve terminals. It was shown that γ-Fe_2_O_3_ nanoparticles decreased the initial rate of ʟ-[^14^C]glutamate uptake (Figure 3a) and increased the ambient level of ʟ-[^14^C]glutamate in the nerve terminal preparations (Figure 3b). One of the reasons that can explain the γ-Fe_2_O_3_-mediated decrease in glutamate uptake is the formation of a protein biocorona from crucial proteins of the plasma membrane immediately after their addition to synaptosomes, which in turn disturbs activity and function of these proteins. In this context, nanoparticle-induced changes of the glutamate transporter function resulted in a decrease in uptake and an increase in the extracellular level of glutamate in nerve cells.

It should be noted that different concentrations of γ-Fe_2_O_3_ nanoparticles were used in the experiments. Nanoparticles within a concentration range of 0.75–3 mg/mL were applied in ʟ-[^14^C]glutamate adsorption experiments in different media without nerve terminals (Figure 2, Figure 5, and Figure 7); a concentration of 1 mg/mL was used in synaptosomal ʟ-[^14^C]glutamate uptake/ambient level experiments (Figure 3); concentrations of 1 µg/mL and 50 µg/mL were used in the fluorimetric experiments on the synaptosomal membrane potential (Figure 4); and a concentration of 0.5 mg/mL was used in laser correlation spectroscopy measurements in biological fluids (Figure 6 and Figure 8). Thus, γ-Fe_2_O_3_ nanoparticles in a concentration range of 1 µg/mL to 1 mg/mL were used in biological experiments and a higher concentration range of 0.75–3 mg/mL was used for adsorption measurements. Due to the overlap of above concentrations, we can conclude that nanoparticle-induced changes in synaptosomal ʟ-[^14^C]glutamate uptake/ambient level resulted from interaction of nerve terminals with the nanoparticles, but not from glutamate/ʟ-[^14^C]glutamate absorption at the surface of the nanoparticles. This is because no glutamate/ʟ-[^14^C]glutamate coatings were registered in the synaptosomal incubation medium. There was also a limit to the nanoparticle concentration in the fluorescence experiments because of poor probe transparency (Figure 4b). Literature data showed that nanoparticles at high concentrations can be applied in biological experiments. For instance, Maier-Hauff et al. used around 31 mg of nanoparticles per cubic centimeter of tissue [35], Zanganeh et al. incubated cells with nanoparticles at a concentration of 30 mg/mL and revealed no direct cytotoxic effects at 3 mg Fe per mL [36].

It was revealed in our study that albumin, a commonly used protein biocorona compound, did not prevent glutamate adsorption at the surface of γ-Fe_2_O_3_ nanoparticles (Figure 7b). This fact should be further investigated to address several questions, namely, whether nanoparticles possess simultaneously a glutamate biocoating and an albumin biocorona or whether the primary biocoating prevents the formation of the albumin biocorona. It was shown that glutamate biocoating was hindered by blood plasma and only 15% of the glutamate biocoating in water was available in blood plasma (Figure 7a). Laser correlation spectroscopy confirmed an increase in nanoparticle size in the synaptosomal incubation medium (the standard salt solution) and a prevention of this increase by blood plasma. Thus, glutamate biocoating of γ-Fe_2_O_3_ nanoparticles in water missed a major amount of glutamate molecules in the nerve terminal incubation medium (Figure 5) and in blood plasma (Figure 7a). The experiments regarding blood plasma and albumin bring insight to one of the essential nanotechnological tasks, that is, the preliminary autologous biocorona formation at the nanoparticle surface before medical application. Autologous biocorona formation is considered to be very promising for advanced nanovesicle technology for cancer treatment [13,37].

Effective magnetic field-mediated glutamate delivery using γ-Fe_2_O_3_ nanoparticles can be used for the modulation of extracellular glutamate homeostasis and synaptic neurotransmission. Also, glutamate plays an important role in mediating the blood–brain barrier function and can be exploited for clinical translation [38]. It was shown that the conjugation of non-permeable drugs with glutamate may improve the brain delivery of the drug [39]. The neuronal release of glutamate modulates the blood–brain barrier function, through activation of *N*-methyl-ᴅ-aspartate (NMDA) receptors [40]. Glutamate increased intracellular calcium levels in endothelial cells and levels of nitrogen oxide (NO) around microvessels. These results can be considered in support of a mechanism of glutamate-induced activation of NMDA receptors in endothelial cells, which leads to calcium signaling and downstream NO production to promote blood–brain barrier permeability [38]. Thus, it may be expected that glutamate-conjugated γ-Fe_2_O_3_ nanoparticles can more easily penetrate the blood–brain barrier than uncoated ones. Glutamate adsorbed on γ-Fe_2_O_3_ nanoparticles in water is expected to be partially released from the nanoparticle surface after contact with biological fluids. Application of magnetic fields can make this a targeted process.

Vice versa, γ-Fe_2_O_3_ nanoparticles can be used (but with lower effectiveness) for glutamate adsorption in the organism followed by its consequent removal using magnetic fields. Glutamate adsorption at the nanoparticle surface and its removal efficiency in blood plasma is only 15% of that in water. It is well-established that high extracellular glutamate levels in the nerve cells lead to excitotoxicity and cell death in stroke and brain trauma. Epilepsy seizures are also accompanied with increased extracellular glutamate concentrations. The balance between the interstitial glutamate concentration in the brain and in the blood was established [41]. Also, an increase in glutamate concentration in blood plasma after stroke episodes was found [42]. In this context, even a low efficacy of γ-Fe_2_O_3_ nanoparticles to adsorb glutamate can be used for the prevention of neurotoxicity in the blood and also in the brain and peripheral nervous system after further technological optimization.

In addition, glutamate is a potential growth factor in tumor development. Glutamate can be removed using γ-Fe_2_O_3_ nanoparticles not only from brain cancer but also from peripheral tissue cancer. It was shown that cells from non-CNS cancers may secrete glutamate into their extracellular environment [5–6,43–45]. The released glutamate can act as a growth factor or/and a signal mediator in tumor tissues [5]. In this context, the formation of a glutamate biocoating at the surface of γ-Fe_2_O_3_ nanoparticles can be potentially useful in stroke, brain trauma, epilepsy, and cancer treatment.

## Conclusion

This study demonstrated that glutamate biocoating is a temporal dynamic structure at the surface of γ-Fe_2_O_3_ nanoparticles. Compounds of the synaptosomal incubation medium that caused a removal of the glutamate biocoating were identified. It was found that blood plasma components other than albumin are responsible for glutamate desorption from the surface of γ-Fe_2_O_3_ nanoparticles. Glutamate-conjugated γ-Fe_2_O_3_ nanoparticles can be used for neurotransmitter delivery for modulation of extracellular glutamate homeostasis and synaptic neurotransmission. Vice versa, these nanoparticles can be used (but with lower efficacy) for glutamate adsorption in different tissues following by its consequent removal with magnetic fields.

## Supporting Information

File 1 Simulation of the spatial structure of a maghemite nanoparticle coated with a blood plasma protein biocorona.
