# Supplementary material for: Transient coating of γ-Fe2O3 nanoparticles with glutamate for its delivery to and removal from brain nerve terminals
Source: Beilstein J Nanotechnol. 2020 Sep 10;11:1381–93. doi: 10.3762/bjnano.11.122 (PMC7492693; doi:10.3762/bjnano.11.122)
Supplement: File 1 — Simulation of the spatial structure of a maghemite nanoparticle coated with a blood plasma protein biocorona. [file Beilstein_J_Nanotechnol-11-1381-s001.pdf]

BEILSTEIN JOURNAL OF NANOTECHNOLOGY

Supporting Information

for

Transient coating of γ-Fe_2O_3 nanoparticles with glutamate for its delivery to and removal from brain nerve terminals

Konstantin Paliienko, Artem Pastukhov, Michal Babič, Daniel Horák, Olga Vasylchenko and Tatiana Borisova

Beilstein J. Nanotechnol. **2020,** *11,* 1381–1393. doi:10.3762/bjnano.11.122

Simulation of the spatial structure of a maghemite nanoparticle coated with a blood plasma protein biocorona

License and Terms: This is a supporting information file under the terms of the Creative Commons Attribution License (https://creativecommons.org/licenses/by/4.0). Please note that the reuse, redistribution and reproduction in particular requires that the authors and source are credited.
The license is subject to the *Beilstein Journal of Nanotechnology* terms and conditions: (https://www.beilstein-journals.org/bjnano)

A maghemite nanoparticle with a diameter of 9 nm was constructed from the original single crystal of a maghemite lattice (**Figure S1**). The model particle has 87 682 ferrum atoms and 108 966 oxygen atoms.

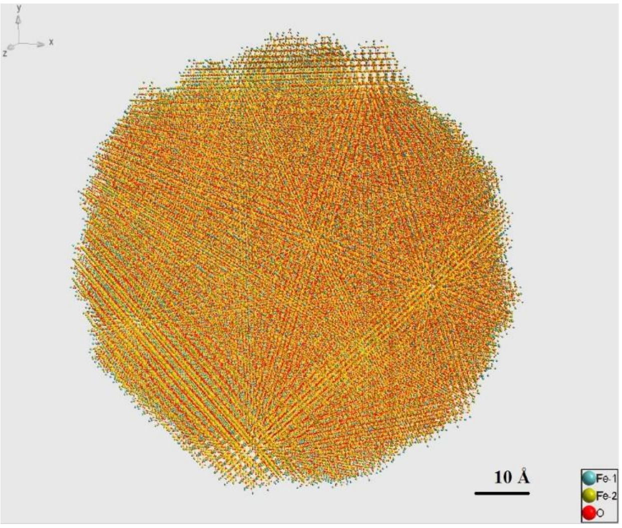

Figure S1: Crystal structure of a maghemite nanoparticle, 9 nm in diameter. Fe-1 is the tetrahedral field ferrum atom, Fe-2 is an octahedral field ferrum atom, and O is an oxygen atom, built with Diamond 4.5.3.

Main proteins of the blood plasma are albumin, fibrinogen and immunoglobulin G. The search of binding sites of protein molecules was performed using the LeadIT 2.3.2 program. Crystal structures of proteins were from the on-line Protein Data Bank (PDB) service. Human albumin molecule (PDB ID: 1E7H) has only one polypeptide chain in its structure [1]. Two specific binding sites, one of the 34 amino acid residues, and the other – 21 residues were detected for this molecule (**Figure S2a,b**).

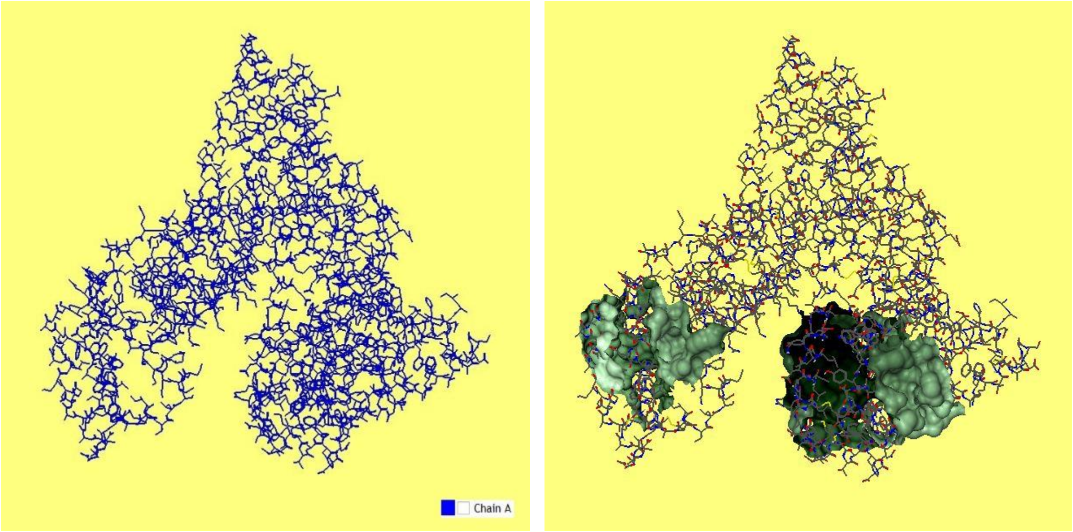

(**a**) (**b**)

Figure S2: Three-dimensional image of the molecular-spatial structure of albumin. (**a**) Prepared structure for analysis without crystalline water (chain A); (**b**) structure with dedicated binding sites.

The fibrinogen molecule (PDB ID: 1MI1J) consists of 6 chains that form pairwise subunits: A and D chains form the α-subunit, B and E – β-subunit, and C and F – γ-subunits of fibrinogen

[2]. Fibrinogen is much larger than a nanoparticle of 9 nm. Therefore, for this molecule only the sites of binding were considered (without further simulation of the nanocomposite formed by several nanoparticles). **Figure S3** showed the image of the molecular structure for further analysis, and the structure after the computer processing.

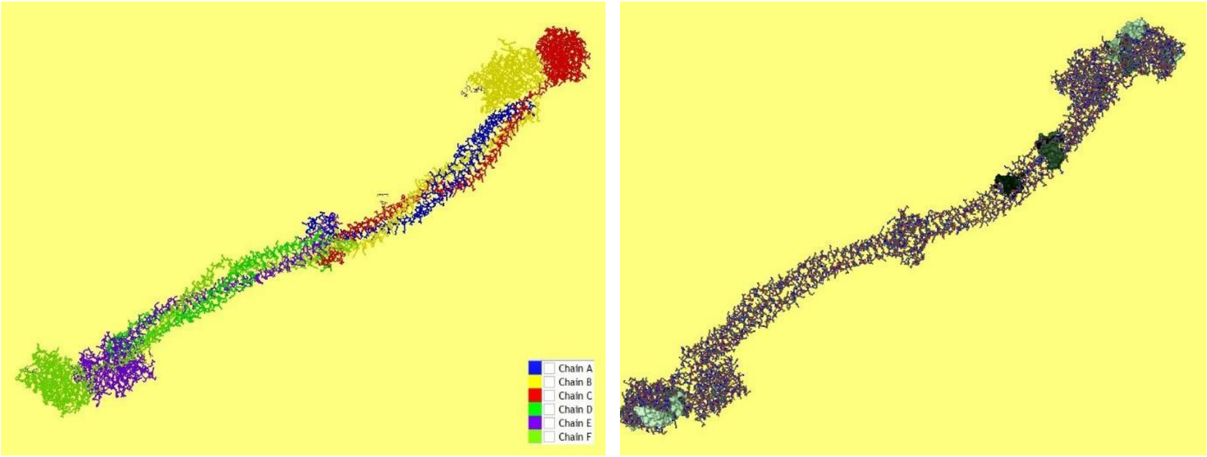

(**a**) (**b**)

Figure S3: Three-dimensional image of the molecular-spatial structure of fibrinogen. (**a**) Structure without crystalline water (chains A, B, C, D, E, F); (**b**) structure with nanoparticle binding sites.

Because of their size, the chains A and D have rather large twisting angles and are constantly in motion, which causes their low ability to attach to the nanoparticle. The sites that are at the ends of molecule have a lot of electronegative amino acid residues that can explain the affinity to the ferrum atoms.

Immunoglobulin G2a, a monoclonal antibody (PDB ID: 1IGT), has antigen-binding sites (chains A and C) in its structure, as well as two H-chains (chain B and D) [3]. IgG can attach to the nanoparticle with the H-chain [4]. As shown by the computer analysis, the IgG molecule has three specific docking sites to the Fe_2O_3 (**Figure S4**).

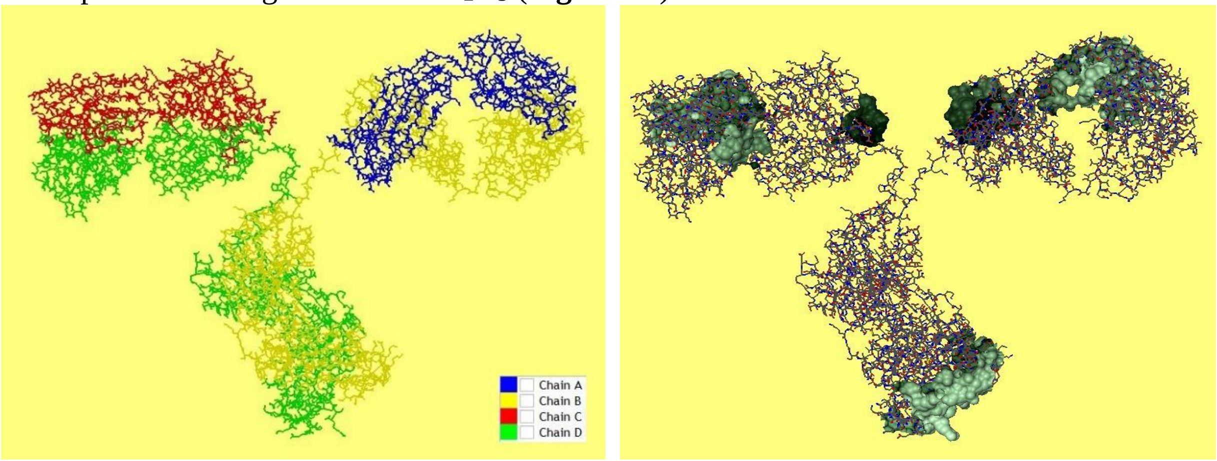

(**a**) (**b**)

Figure S4: A three-dimensional image of the molecular-spatial structure of human IgG. (**a**) Structure without crystalline water (chains A, B, C, D); (**b**) probable sites for nanoparticle binding.

The programmed calculation of the doping of the ligand with the receptor should include changes in the dynamic transformation of the molecule, such as shifts and turns, as well as internal changes in the structure, including the rotation of the twisting angle. Each of these steps in the

conformational space of the ligand constantly changes the overall energy level of the system. Thus, the total energy of the system is calculated after each movement [5]. To construct a graphic model of a biomodified nanoparticle, data from a computer analysis of the most probable domains of binding of each protein to the surface of the maghemite nanoparticle were used. The construction of the graphic model was performed only for the rigid layer of biocorona, that is, computer simulation of the direct interaction of the protein as a ligand compound with a spherical surface layer of iron oxide as a receptor structure. The simulation of the fibrinogen interaction with nanoparticles was not carried out because of the over-sized model particle. Doping of protein molecules into nanoparticles and subsequent graphical optimization were performed using the programs ArgusLab 4.0.1 and Material Science Suite 2015 (**Figure S5**). The number of protein molecules forming the biocorona layer amounted to seven albumin molecules and three IgG molecules.

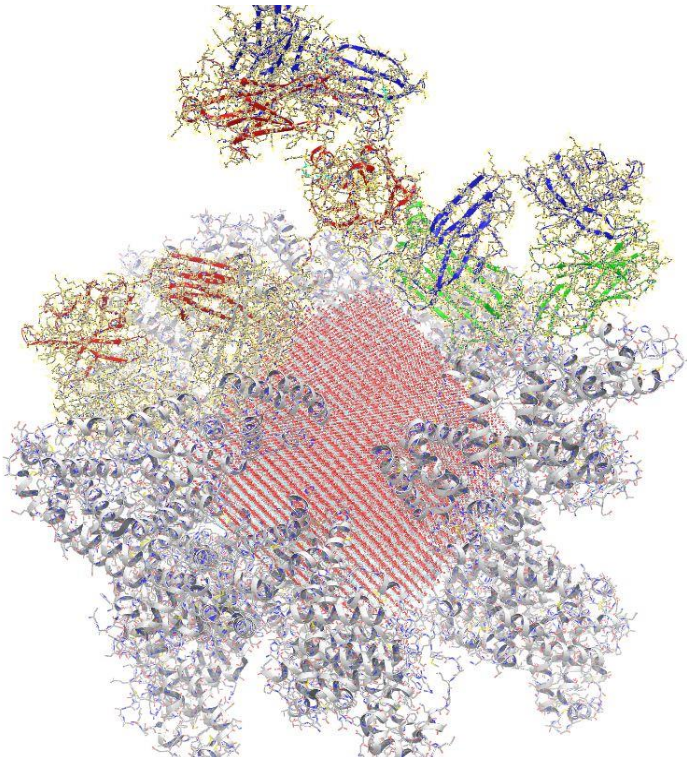

Figure S5: Three-dimensional model of a maghemite nanoparticle biomodified with plasma proteins. The nanoparticle on which immunoglobulin G (yellow) and albumin (white helices) molecules are adsorbed.

References

(1) Bhattacharya, A. A.; Grüne, T.; Curry, S. *J. Mol. Biol.* **2000,** *303,* 721–732. doi:10.1006/jmbi.2000.4158

(2) Yang, Z.; Kollman, J. M.; Pandi, L.; Doolittle, R. F. *Biochemistry* **2001,** *40,* 12515–12523. doi:10.1021/bi011394p

(3) Harris, L. J.; Larson, S. B.; Hasel, K. W.; McPherson, A. *Biochemistry* **1997,** *36,* 1581–1597. doi:10.1021/bi962514+

(4) Lara, S.; Alnasser, F.; Polo, E.; Garry, D.; Lo Giudice, M. C.; Hristov, D. R.; Rocks, L.; Salvati, A.; Yan, Y.; Dawson, K. A. *ACS Nano* **2017,** *11,* 1884–1893. doi:10.1021/acsnano.6b07933

(5) Taylor, R. D.; Jewsbury, P. J.; Essex, J. W. *J. Comput. Chem.* **2003,** *24,* 1637–1656. doi:10.1002/jcc.10295
